# Knowledge and practice of infection control – in the NDM1 era

**DOI:** 10.1186/2047-2994-4-S1-P118

**Published:** 2015-06-16

**Authors:** V Lakshmi, A Ghafur, K Mageshkumar, C Karupusamy

**Affiliations:** 1 Infectious Diseases, Apollo Speciality Hospitals, Chennai, India; 2 Infection Control, Apollo Speciality Hospitals, Chennai, India

## Introduction

India has reported one of the highest rates gram negative resistance in the world. Knowledge on infection control and the translation of this into practice is of paramount importance for the performance of health care institutions.

## Objectives

To assess the knowledge, attitude and practice on infection control practices in an ICU in an oncology, BMT and neurosurgical centre in South India.

## Methods

This is a descriptive and observational study. A questionnaire was distributed amongst the health care workers (HCW), which included topics related to various infections control practices. Subsequent to this an observational study was also done on the same participants.

## Results

Sixty nine respondents participated in the survey which predominantly included the nurses (60). 72.4% of the respondents had 1-5 years of experience in the hospital. Knowledge regarding PPE and Biomedical waste disposal based on questionnaire (Q in %) was 98.5 and compliance by Observational (O in %) was 92.5. Adherence to VAP bundle Q 97.1, O- 86.9, adherence to CRBSI bundle 92.7,O -86.9, oral hygiene for VAP prevention Q 86.9, O-83. Knowledge on high end antibiotic Q-92.7, O (helped in tracking this ) -92.7%, use of alcohol based hand rub Q-98.5,O-94.2, five moments of hand hygiene Q and O 88.5, 6 steps of hand hygiene Q-92.5, O-65%.

## Conclusion

There is a very good concordance between knowledge and practice of most participants in most of the components, except in the practice of performing all the six steps of hand hygiene where there was a discrepancy, though the rate was still very good. Most of the participants were nurses and so good overall compliance rate is expected. A study with active participation of doctors will reveal the true concordance between knowledge and practice of infection control, especially hand hygiene practices. Such audits will bridge the gap between awareness and practices.

## Disclosure of interest

None declared.

